# Supplementary material for: Effect of smoking cessation interventions on abstinence and tuberculosis treatment outcomes among newly diagnosed patients: a randomized controlled trial
Source: Microbiol Spectr. 2024 Feb 22;12(4):e03878-23. doi: 10.1128/spectrum.03878-23 (PMC10986535; doi:10.1128/spectrum.03878-23)
Supplement: Meta analysis — Forest plot showing the pooled relative risk of abstinence among TB patients with smoking cessation intervention compared to control group (no intervention) along with weights of individual studies [file spectrum.03878-23-s0001.docx]

Search strategy and results obtained using keywords via Ovid

|  | SEARCHES | RESULTS |
| --- | --- | --- |
| 1. | smok* | 171438 |
| 2. | limit 1 to human | 628423 |
| 3. | tuberculosis | 509592 |
| 4. | limit 3 to human | 349887 |
| 5. | 2 and 4 | 5769 |
| 6 | remove duplicates from 5 | 4248 |


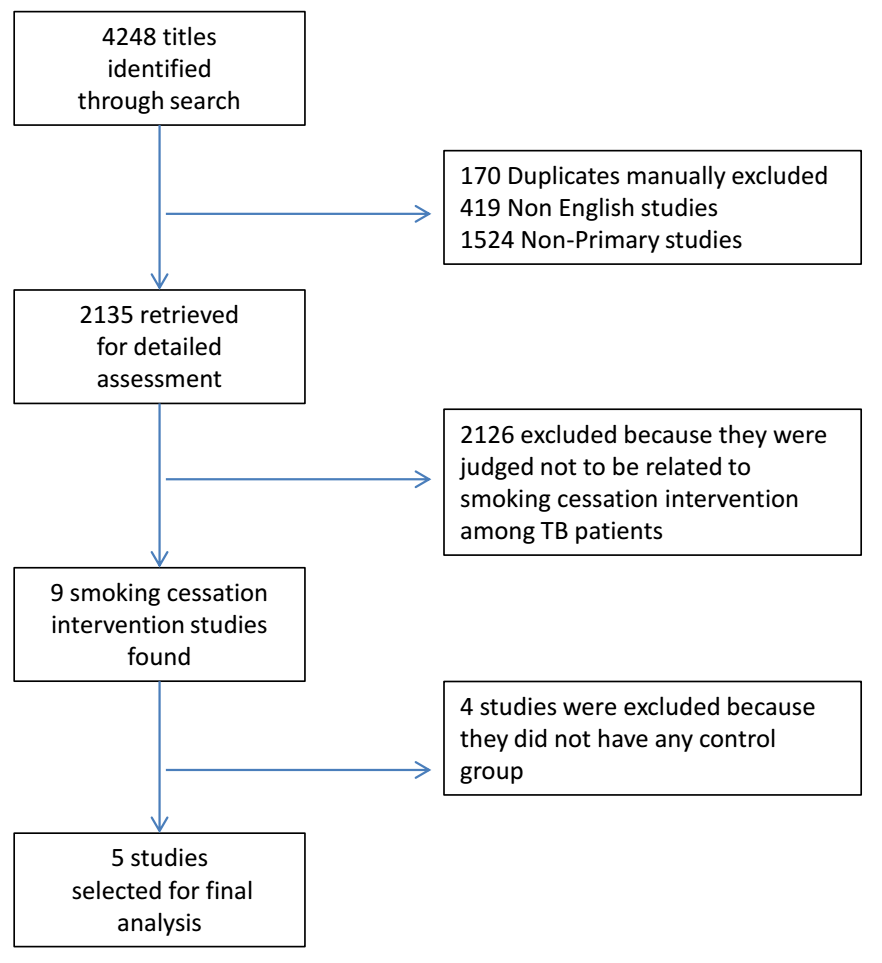
 Study attrition diagram
