# Supplementary figures and images for: Effect of smoking cessation interventions on abstinence and tuberculosis treatment outcomes among newly diagnosed patients: a randomized controlled trial
Source: Microbiol Spectr. 2024 Feb 22;12(4):e03878-23. doi: 10.1128/spectrum.03878-23 (PMC10986535; doi:10.1128/spectrum.03878-23)

Supplementary File 2


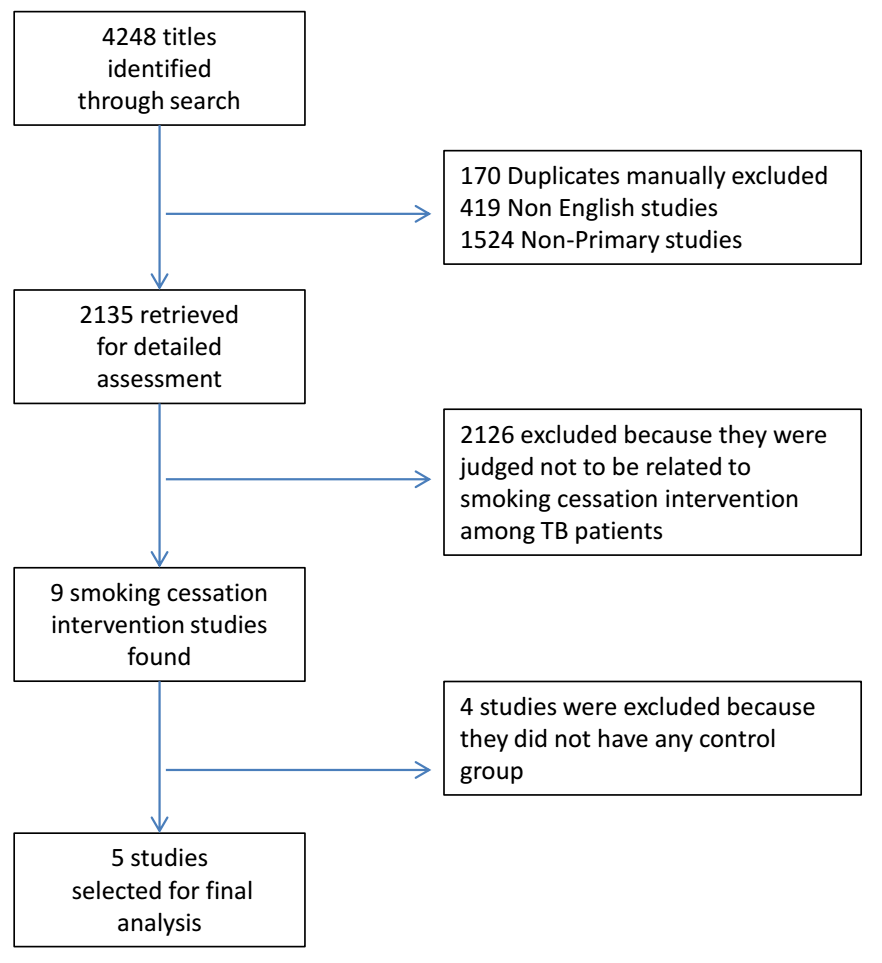


Study attrition diagram

Supplement: Attrition diagram — Study attrition diagram for systematic review and meta analysis. [file spectrum.03878-23-s0002.docx]
