# Supplementary material for: Effect of smoking cessation interventions on abstinence and tuberculosis treatment outcomes among newly diagnosed patients: a randomized controlled trial
Source: Microbiol Spectr. 2024 Feb 22;12(4):e03878-23. doi: 10.1128/spectrum.03878-23 (PMC10986535; doi:10.1128/spectrum.03878-23)
Supplement: Search strategy — Study search strategy using OVID for meta analysis. [file spectrum.03878-23-s0003.docx]

Supplementary File 3


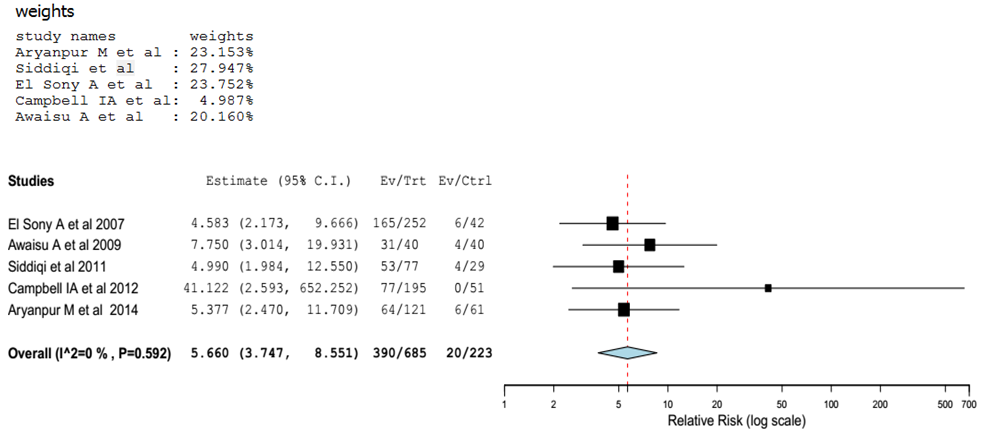


Forest plot showing the pooled relative risk of abstinence among TB patients with smoking cessation intervention compared to control group (no intervention) along with weights of individual studies
